# Intelligent Perception-Based Cattle Lameness Detection and Behaviour Recognition: A Review

**DOI:** 10.3390/ani11113033

**Published:** 2021-10-22

**Authors:** Yongliang Qiao, He Kong, Cameron Clark, Sabrina Lomax, Daobilige Su, Stuart Eiffert, Salah Sukkarieh

**Affiliations:** 1 Australian Centre for Field Robotics (ACFR), Faculty of Engineering, The University of Sydney, Sydney, NSW 2006, Australia; he.kong@sydney.edu.au (H.K.); s.eiffert@acfr.usyd.edu.au (S.E.); salah.sukkarieh@sydney.edu.au (S.S.); 2 Livestock Production and Welfare Group, School of Life and Environmental Sciences, Faculty of Science, The University of Sydney, Sydney, NSW 2006, Australia; cameron.clark@sydney.edu.au (C.C.); sabrina.lomax@sydney.edu.au (S.L.); 3 College of Engineering, China Agricultural University, Beijing 100083, China; sudao2020@outlook.com

**Keywords:** cattle behaviour, lameness detection, precision livestock farming, intelligent perception, cattle welfare

## Abstract

**Simple Summary:**

Cattle lameness detection as well as behaviour recognition are the two main objectives in the applications of precision livestock farming (PLF). Over the last five years, the development of smart sensors, big data, and artificial intelligence has offered more automatic tools. In this review, we discuss over 100 papers that used automated techniques to detect cattle lameness and to recognise animal behaviours. To assist researchers and policy-makers in promoting various livestock technologies for monitoring cattle welfare and productivity, we conducted a comprehensive investigation of intelligent perception for cattle lameness detection and behaviour analysis in the PLF domain. Based on the literature review, we anticipate that PLF will develop in an objective, autonomous, and real-time direction. Additionally, we suggest that further research should be dedicated to improving the data quality, modeling accuracy, and commercial availability.

**Abstract:**

The growing world population has increased the demand for animal-sourced protein. However, animal farming productivity is faced with challenges from traditional farming practices, socioeconomic status, and climate change. In recent years, smart sensors, big data, and deep learning have been applied to animal welfare measurement and livestock farming applications, including behaviour recognition and health monitoring. In order to facilitate research in this area, this review summarises and analyses some main techniques used in smart livestock farming, focusing on those related to cattle lameness detection and behaviour recognition. In this study, more than 100 relevant papers on cattle lameness detection and behaviour recognition have been evaluated and discussed. Based on a review and a comparison of recent technologies and methods, we anticipate that intelligent perception for cattle behaviour and welfare monitoring will develop towards standardisation, a larger scale, and intelligence, combined with Internet of things (IoT) and deep learning technologies. In addition, the key challenges and opportunities of future research are also highlighted and discussed.

## 1. Introduction

Livestock production is the second largest supplier of food for human consumption, after vegetable/cereal agriculture. The livestock sector contributes up to 50% of the global agricultural gross domestic product and supports the livelihoods and food security of almost 1.3 billion people in developing countries [1]. The increasing demand of livestock products is the result of human population growth, urbanisation, and growing incomes. The United Nations Food and Agriculture Organisation predicts a 60% increase in demand for animal products (i.e., meat, milk, and eggs) by 2050 [2]. To meet the rising global demand for animal products, the livestock industry will need to improve its efficiency and operations to enhance productivity per animal. Along with this, there are also growing concerns about wastewater, air pollutants caused by animal manures, CO${}_{2}$ emissions, and animal ethical issues. However, rising labour and maintenance costs alongside the increasing number of animals per farm have reduced the levels of individual animal care [3,4]. In addition, some animal farming problems such as high levels of lameness, inefficient use of resources, reduced species diversity, reproduction, and cows’ short lifespan also need to be considered.

In order to provide sufficient care for each individual animal to increase productivity and yield, automatic livestock monitoring and management technologies are needed. With the ability to increase efficiency by shifting the focus towards the individual animal, precision livestock farming (PLF) has attracted much attention from both governments and the industry in recent years [5]. Conceptually, PLF involves the integration and interpretation of relevant sensor information to enable the management of individual animals through continuous real-time monitoring of their health, behaviour, production/reproduction, and environmental impact [6,7]. Through technologies such as machine learning and Internet of Things (IoT, i.e., the interconnection between computing devices via the Internet), decision making in PLF can be better managed by fusing and analysing different sensor data streams, thereby reducing operational costs and improving animal health and welfare while increasing productivity, yield, and environmental sustainability. The intelligent perception and analysis of individual animal’s behaviours, welfare, and production is fundamental for improving sustainable production systems [4]. The concept of intelligent perception for animal monitoring was proposed by [8] and refers to perceptive animal bio-response from the animal–environment interaction using multi-senor data and the ability to apply adaptive learning to analyse animal welfare and health status [9]. In recent years, sensors such as cameras, microphones, 3D accelerometers, temperature sensors, glucose sensors, and technologies such as deep learning and the IoT make it increasingly feasible to model, monitor, and control animal bio-response and to provide accurate feedback to the farmer. Grounded on this basis, combined with the development of a Decision Support System (DSS) or expert systems, intelligent perception technologies can make large-scale animal husbandry more cost-effective, efficient, and sustainable [10,11].

Cattle lameness is a key factor for reduced performance on many farms. The timely detection of lameness is important for providing effective and inexpensive treatment and for preventing future ailments [12]. Meanwhile, cattle behaviour is an important indicator for animals’ health and welfare, which influence the quantity and quality of cattle products [13]. However, traditional lameness detection and behaviour recognition approaches are time-consuming and labour-intensive, resulting in major concerns on farms. Here, we focus on intelligent perception and analysis technologies relevant for the following two main tasks: (1) lameness detection; (2) cattle behaviour recognition and analysis. In this work, we summarise and analyse recent work in the above areas and discuss future research, and developmental opportunities and challenges. For an overview of existing technologies used for cattle identification, body condition score evaluation, and weight estimation, we refer the reader to [14].

**Figure 1:**
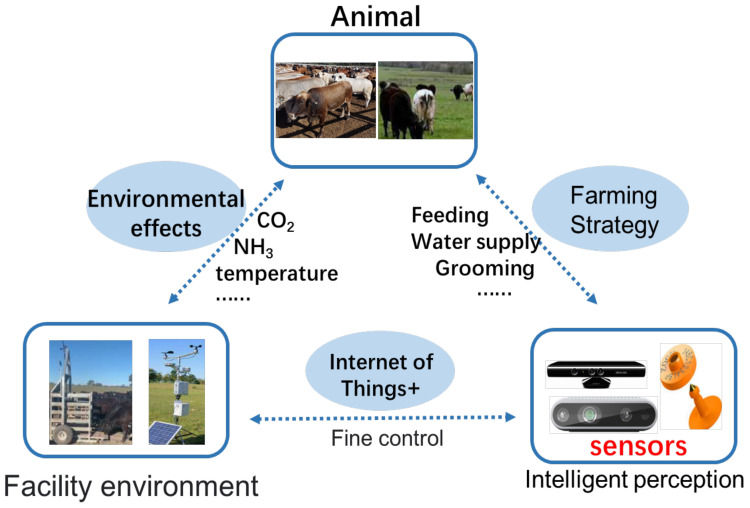
The framework of intelligent perception-based animal farming.

In this study, we focus on intelligent perception techniques for precision beef cattle farming. We also partially review existing studies on dairy cattle. This is mainly because some technologies and methods could be used to resolve common concerns in both beef and dairy cattle. Therefore, we focus on beef cattle in this work and discuss some references corresponding to dairy cows. A general intelligent perception-based animal farming process is presented in Figure 1. In this framework, the animal welfare measurement with relevant smart sensors is the key part, and a DSS utilises the former information to manage farming and environment protocols [15,16,17].

## 2. Cattle Lameness Detection and Scoring

### 2.1. Cattle Lameness

Lameness is a prevalent health issue in cattle production, impacting both animal welfare and livestock productivity. Painful disorders in the locomotor system result in the animal modifying its gait and posture to minimise pain, which is observed as impaired motion, or non-standard gait or posture [18,19,20]. The main causes of lameness include Hoof lesions [12], limb lesions, or locomotor deficiencies [21]. Lameness in cattle restricts locomotion and movement and leads to reduced milk production, lower fertility, and higher culling rates [22]. As a consequence, it is the third most costly health issue after reproduction issues and mastitis in the dairy industry [23]. Lameness affects not only animal welfare but also yield and profit. In addition, due to its high prevalence on farms, lameness is regarded as a major health and economic concern in modern cattle farming. As such, the detection of lameness in an accurate and timely manner is of great significance [24,25]. However, the cause and prevalence of lameness vary between production systems (pastures and barns) and farm management but lameness is typically found in between 10% and 30% of the herd [26].

### 2.2. Manual Cattle Lameness Detection Approaches

Lameness can be detected manually by visually observing behavioural changes as lame animals reduce their speed, change their pace, arch their backs, and drop their heads during walking [27]. A manual Locomotion Scoring System (MLSS) is a systematic method of assessing lameness [28] with the locomotion of an animal scored on an ordinal scale by humans who watch for specific locomotion traits [28]. Sprecher et al. [22] and Winckler and Willen [29] scored cow lameness by considering the step consistency, step size, and load of a dairy cow’s gait. More recently, Thomsen et al. [30] used the threshold judgment method to make the lameness scoring system reliable. It should be remarked that the score from MLSS is subjective, as it is influenced by the examiner’s experience and perceptions [31,32]. Moreover, as the intensity and scale of cattle farming increase, farmers tend to have less time to conduct manual lameness assessments.

### 2.3. Automatic Cattle Lameness Detection Approaches

In recent years, electronic sensors and artificial intelligence techniques have been introduced into the livestock industry for lameness detection. With these emerging technologies, lameness detection can be performed in a more timely and accurate manner [32]. Advances in sensor technologies and other fields have been adapted to automatic lameness detection. For example, there is recent research dedicated to detecting lameness automatically with Automatic Locomotion Scoring Systems (ALSSs) [19,33]. In contrast to MLSSs, ALSSs could provide a more objective, consistent lameness assessment [20]. Locomotion was classified into lameness levels in recent studies using more advanced metrics such as body movement pattern [34], gait [35], and step frequency [36].

The most popular sensors used in automatic cattle lameness detection include force platforms [37], two-dimensional (2D) and three-dimensional (3D) cameras [38], and on-limb accelerometers [39]. Measurements from these sensor are the input to algorithms to compute lameness traits such as step overlap [33,40] and back curvature [38].

According to [19,27], automatic lameness detection approaches can be categorised into three classes: kinetic (measuring forces involved in locomotion), kinematic (measuring limb trajectories in space and time, and related specific posture variables), and indirect measurement techniques (measuring behavioural or production variables). An overview of the most popular lameness detection methods and the corresponding locomotion traits observed is given in Table 1.

#### 2.3.1. Kinetic Approaches

Cattle lameness can be detected through an analysis of cattle motion and the causes of motion such as forces, or translational and rotational torque. This is known as the kinetic framework. In kinetic approaches, hoof forces or weight distribution when cattle are walking or standing, respectively, are often used to evaluate locomotion scores. For example, Liu et al. [37] and Dunthorn et al. [41] used a force-plate to measure the leg force and applied logistic regression to detect cattle lameness. It should be noted that in early work on kinetic approaches, only vertical ground reaction forces were considered for lameness detection. In recent years, ground reaction forces in three dimensions have been measured and utilised for lameness detection [38,41].

Deep learning approaches have also gained some recent interest. For example, Wu et al. [42] proposed a lameness detection approach for dairy cows based on YOLOv3. It is worthwhile mentioning that, in real practical experiments, the lameness detection accuracy of kinetic approaches is affected by the cattle hoof position on the weighing units during measurement [43] or by the walking speed of cows [44].

**Table 1:** Main studies on lameness detection.

Work	Sensor	Dataset Size (Cattle Number)	Traits	Model	Automation Level	Results
kinetic						
Liu et al. [37]	force plate	346	vertical kinetic	logistic regression	medium	sensitivity = 51.92%
Dunthorn et al. [41]	3D force-plate	85	leg force	logistic regression	medium	sensitivity = 90.0%
Nechanitzky et al. [45]	weighing platform	44	weight and laying time	logistic regression	medium	sensitivity = 81.0%
Chapinal et al. [36]	camera weighing platform	57	step frequency laying time, weight	logistic regression	high	Area under the curve = 83.0%
Chapinal and Tucker [46]	camera weighing platform	257	step number and gait	statistic analysis	high	sensitivity ≥ 0.96
Zillner et al. [47]	clock	53	walking speed	analysis of variance	low	sensitivity = 71.43%
kinematic						
Van Nuffel et al. [48]	gaitwise system	61	gait	linear discriminant	medium	sensitivity = 88.0%
Pluk et al. [40]	camera	85	step overlap	regression model	medium	$R_{2}$ = 80.90%
Poursaberi et al. [49]	camera	156	back curvature	image analysis	high	accuracy = 96.7%
Poursaberi et al. [50]	camera	1200	posture and movement	image analysis	high	accuracy = 92.0%
Viazzi et al. [34]	camera	90	posture and movement	image analysis	high	accuracy = 76.0%
Viazzi et al. [38]	3D camera	273	back posture	decision tree	high	accuracy = 90.0%
Van Hertem et al. [35]	3D camera	186	gait	logistic regression model	high	accuracy = 60.2%
Van Hertem et al. [51]	3D camera	208	back posture	binary GLMM	high	accuracy = 79.8%
Wu et al. [42]	camera	50	step size	long short-term memory	high	accuracy = 98.57%
Zhao et al. [12]	camera	98	leg swing	decision tree classifier	high	sensitivity = 90.25%
Beer et al. [52]	Camera	63	gait	logistic regression model	medium	sensitivity = 90.2%
Jiang et al. [53]	camera	30	walking characteristics	double normal distribution statistical	high	accuracy = 93.75%
Jabbar et al. [54]	3D camera	22	height variation	support vector machine	high	accuracy = 95.7%
Kang et al. [55]	camera	100	supporting phase	data analysis	high	accuracy = 95.7%
Piette et al. [56]	camera	209	back posture	threefold cross validation	high	accuracy = 82.0%
In direct						
De Mol et al. [57]	3D accelerometers	100	lying time	dynamic linear model	high	sensitivity = 85.5%
Kamphuis et al. [58]	pedometers, weigh scales milk meters	292	live weight, steps milk yield	dynamic linear model	high	sensitivity = 80.0%
Miekley et al. [59]	milk meter pedometers	338	milk yield feeding patterns	principal component analysis	high	sensitivity = 87·8%
Kramer et al. [60]	milk meter neck transponders	125	milk yield and activity	fuzzy logic model	high	sensitivity ≥ 70.0%
Chapinal et al. [44]	camera	153	gait score, walking speed lying behaviour	discriminant analysis	high	sensitivity = 67.0%
Garcia et al. [61]	automatic milking system	88	milk yield and activity variables	discriminant analysis	high	sensitivity ≥ 80.0%
Wood et al. [62]	Infrared thermometry	153	foot temperature	linear regression	high	coefficient = 62.3%
Lin et al. [63]	infrared thermometers	990	foot-surface temperatures	linear regression	high	sensitivity = 78.5%
Jabbar et al. [54]	3D camera	22	shape index and curvedness	SVM	high	accuracy = 95.7%
Taneja et al. [64]	camera	150	step count, lying time, swaps	K-Nearest Neighbours	high	accuracy = 87.0%
Jiang et al. [53]	camera	30	pixel distribution characteristics	statistical model	high	accuracy = 93.75%

Note: SVM means support vector machine; GLMM means Generalised linear mixed model; Gaitwise is a pressure-sensitive measure system.

#### 2.3.2. Kinematic Approaches

Unlike kinetic approaches that try to measure ground reaction forces, kinematic approaches focus on kinematic variables (i.e., how the cattle move spatially and temporally) [20]. In other words, the kinematic approaches only study the motion itself without considering the cause of the motion.

In kinematic approaches, different techniques can be used to obtain variables of locomotion such as step size, step length, height, and back curvature [40,48,65]. In general, kinematic gait variables can be computed based on hoof location pattern. For example, Pluk et al. [65] used a pressure sensitive mat-based Gaitwise system to measure the hoof location with the vertical reaction force and time.

Image/video processing and analysis have also been used for cattle lameness detection, where the recorded cattle videos are transformed into images sequences for kinematics extraction [65]. Here, the hooves, limb joints, and withers were tracked using attached reflective markers, and the kinematic gait parameters (e.g., stride duration, stance duration, and swing duration and hoof speed) were calculated to find ulcers. Moreover, back postures extracted from video frames were used for automatic lameness detection in [49], where a binary classification from a back arch metric resulted in a sensitivity of 100%, a specificity of 97.6%, and correct classification rates in the order of 96.5%. Furthermore, Beer et al. [52] evaluated the feasibility of the newly described parameters (e.g., “calculated walking speed” and “lying bout duration”) of cow gait for the early detection of lameness.

Other techniques use cattle limbs attached accelerometers to measure the acceleration of legs while cattle are walking [66]. Based on the collected measurements, a ratio of acceleration variance and a ratio of wavelet detail between the left and right limbs can be used for lameness detection.

#### 2.3.3. Indirect Approaches

Some variables not directly related to lameness or motion have also been used for lameness detection. The methods in this category are usually called indirect approaches [58,67,68]. Generally speaking, these approaches use sensors to measure behavioural (e.g., lying, standing, and walking time) and production variables (e.g., milking order and milk yield) for final lameness detection [61]. For example, ALSSs in Buisman et al. [25] relied on the use of on-cow sensors such as accelerometers to detect alterations in behaviour, such as the duration of lying or standing bouts, and the total time lying down or standing per day. In fact, lying time is a commonly used behaviour metric in several works [69,70,71], while ALSSs is based on production information and mainly focuses on the live weight, or milk yield and collection time. The production data may be obtained by combining off-cow sensors such as milk meters or weight scales [58].

Miekley et al. [59] proposed a lameness detection method based on pedometer activity and feeding patterns. Kramer et al. [60] used the milk yield and feeding behaviour to predict lameness with the aid of fuzzy logic and achieved specificity in the range of 75% for 125 cows. Gardenier et al. [20] used Faster R-CNN to detect hooves and carpal/tarsal joints to obtain individual trajectories per limb. Jiang et al. [68] learned video representations using neural networks with single-stream long-term optical flow convolution and achieved 98.24% lameness behaviour recognition accuracy with a speed of 564 FPS (1.77 ms/image).

Garcia et al. [61] used the milking, feeding, and behavioural parameters to predict lameness with a partial least squares discriminant analysis model. In that work, a binary lameness classification reached 77% and 83% for parity in two cows. To improve lameness detection performance, in Ishihara et al. [72], a multivariate model combining several variables such as body posture, day-time activity, rear back angle, walking speed, milk yield, and milk flow rate was proposed.

**Figure 2:**
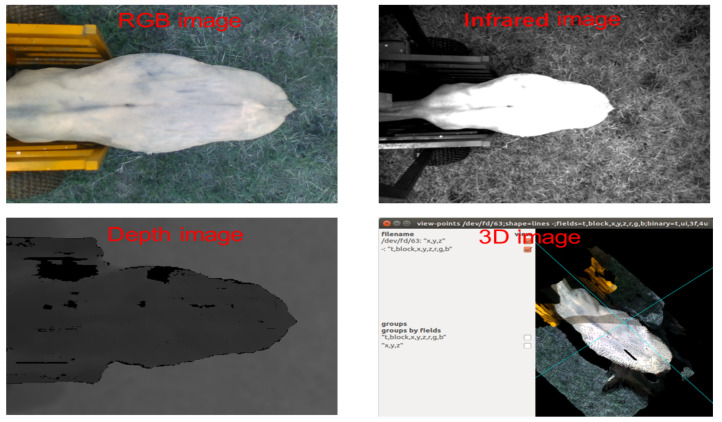
Examples of acquired images from an Intel RealSense D435 camera.

Additionally, 2D cameras and 3D sensors have gained popularity in lameness detection [49]. In these approaches, after image acquisition (Seen in Figure 2), visual features (such as uneven gait and back arch) were extracted to build a lameness detection model. Viazzi et al. [38] compared the 2D and 3D camera systems for cow lameness detection and found that both 3D and 2D camera can achieve more than 90% accuracy. In general, compared with 2D camera, 3D cameras obtain more comprehensive information and are more suitable for long-term observation and data collection for lameness detection. However, the processing of 3D information is complex and time-consuming due to its larger amount of data.

In Song et al. [33], trackway information containing hoof locations in the real world and its corresponding times in a video were calculated for automatic detection of lameness. Van Hertem et al. [51] used a 3D video recording system to automatically quantify the back posture of cows and achieved 79.8% lameness classification accuracy using generalised linear mixed models (GLMM). Jabbar et al. [54] proposed a nonintrusive lameness detection method in dairy cows using 3D video and achieved an overall lameness classification accuracy with 95.7%. Zhao et al. [12] developed an automatic system for scoring the locomotion of cows, quantified the movement patterns of cows for classifying lameness using the features extracted from movement analysis, and achieved 90.18% accuracy.

Recently, Jiang et al. [53] proposed a double normal background statistical model for lameness detection using side-view images and achieved 93.75% detection accuracy. Piette et al. [56] proposed a lameness monitoring algorithm based on back posture values derived from a camera for individual cows and tuned the deviation thresholds and the quantity of the historical data being used. Taneja et al. [64] developed an end-to-end approach that leverages fog computing and K-Nearest Neighbours techniques to identify lame cattle and achieved 87% accuracy for an early lameness detection window of 3 days before visual signs.

Apart from traditional 2D and 3D cameras, thermal infrared cameras have also been used to check hoof temperatures for cattle lameness detection [73]. This is based on the fact that hoof lesions and infection can change the hoof surface temperature due to increased blood flow. Hence, when a cow’s hoof is damaged, the surface temperature increases [74].

For example, Lin et al. [63] proposed a lameness detection approach using infrared thermometers. They analysed the ambient-temperature-adjusted foot-surface temperatures and temperature differences between the hind feet of individual cows to optimise lameness detection. According to their results, the optimal threshold was 23.3 ${}^{\circ }$C with 78.5% sensitivity and 39.2% specificity. However, given the fact that different hoof positions result in varying temperatures, the selection of the threshold value still needs further study. Nevertheless, infrared thermography has great potential as an early diagnostic method for lameness and can compensate for 2D or 3D lameness detection because early lameness motor characteristics may not be as significant [75].

### 2.4. Limitations of Automated Lameness Detection Systems

The availability of low cost and validated automatic lameness detection systems makes monitoring animal lameness behaviour quite feasible. However, the majority of lameness detection systems are still in the research phase and have not yet been commercialised and implemented under field conditions [76].

The use of ALSSs can be influenced by many factors (e.g., experiences of users, the space limitation, and the investment budget). Investment cost, product efficiency, maintenance complexity, robustness, and equipment application ability are the main factors to consider when choosing the type of automatic lameness detection system [27]. A good lameness detection system should be combined with existing farm infrastructures (e.g., weighing platform) and should allow the manager to regularly (e.g., twice or three times a day) check the data. The fusion of different sensors’ measurements such as feeding, milking, and grooming data has the potential to improve the accuracy of lameness detection at an early stage [53].

In terms of automated lameness detection methods, a combination of multiple methods can potentially further improve the robustness and accuracy of detection because it is difficult to detect all lame cows accurately based on only a single feature [64]. Meanwhile, both temporal and spatial characteristics from data can be unified to improve performance of lameness detection [77]. High quality data should be given to lameness detection systems to help farmers make decisions and to provide some early warnings. In addition, parts of lameness detection also rely on the recognition of behaviours such as walking, lying, and feeding; therefore, a comprehensive behaviour recognition and analysis is also helpful in some cases.

### 2.5. Cattle Behaviours

Cattle behaviour mainly refers to the animals’ continuous interaction with the environment and the way they express themselves. Hence, it is a valuable indicator in assessing the health and welfare of animals [78]. The behaviour of domestic cattle has evolved over a long time, initially in response to their domestication [79]. According to recent research, the main cattle activity behaviours in PLF can be classified into grazing, exploring, grooming, mounting, ruminating, lying, walking, standing, and aggressive behaviour [80]. Measuring and assessing the behaviour of livestock is important as it can be used to indicate their pain feeling [81], lameness [67], and welfare status [82]. When animals are ill, their behaviour changes include a decrease in exploratory activity, reproductive activity, food and water intake, grooming, and other social behaviours. Hence, monitoring and analysing changes in behavioural activity could provide useful information for timely management decisions to optimise animal performance, genetic selection and breeding, welfare, and environmental outcomes [83]. In Table 2, the descriptions of some main cattle behaviours are given.

Especially, grazing is an important behaviour from an economic and welfare point of view in PLF [80]. Lying behaviour is a parameter frequently quantified by precision dairy monitoring technologies, since the time that a cattle spends lying down can indicate comfort, welfare, and health changes in an animal [84,85]. Mounting behaviour is the most widely used indicator of reproductive behaviour for estrus detection [86]. Aggressive behaviour can be observed during feeding times when animals compete for food, water, or other resources. There is also some association between aggressiveness and a high level of feeding in a half-open feedlot production system, as investigated in [87].

Recent progress towards cattle behaviour monitoring and analysis can be classified into three different categories: the first category only focuses on behaviour detection, the second category is long-term behaviour monitoring and detection, and the final category is automatic behavioural changes detection and quantification based on long-term behaviour monitoring [88]. Currently, most existing results in the literature focus on the second category—monitoring behaviour over time, with few reports about the third category—detection and quantification of behavioural changes.

### 2.6. Manual Approaches for Cattle Behaviour Monitoring and Recognition

The traditional human observation method for cattle behaviour recognition is time-consuming [89]. For example, Geers et al. [90] reported that the time required for mounting behaviour detection accounts for 30% of the labour involved in commercial farming. Sambraus et al. [91] mentioned that continuous monitoring of mounting behaviour results in 20% of oestrus being undetected. Moreover, recognising individual cattle in a large herd for key management decisions such as the identification of estrus is too labour-intensive [92].

### 2.7. Automatic Approaches for Cattle Behaviour Monitoring and Recognition

Recently, the increasing availability of sensors and machine learning technologies makes automated monitoring and recognition of animal behaviour practicable [93,94]. Sensors that can provide information about animal behaviour can be classified as contact and non-contact ones. On the one hand, contact sensors are usually fitted on (or sometimes in) the animal, for example, tags, collars, global Positioning System (GPS), accelerometer, pedometers, and magnetometer, etc. [95]. On the other hand, non-contact sensors such as camera and LiDAR are cheap, easy, non-stressful, and noninvasive methods. Moreover, non-contact sensors can be adapted to different animals, in both indoor and outdoor situations, using the animals’ natural features (e.g., shape, colour, and movement) for monitoring their behaviours [96].

It should be remarked that an automatic activity monitoring system needs to allow for recording in the animals’ normal environment without influencing the animals’ behaviour. Additionally, cattle can vary in size and shape (spatial), and over a period of time (temporal). Therefore, to collect behavioural phenotypic information, temporal or spatial features (e.g., velocity, acceleration, speed, shape, and contour) can be extracted from sensor data for behaviour recognition.

The concept of features should also include external factors such as temperature and air quality. In addition, the distribution time of feed and drinking also contains useful information that can explain the current conditions influencing cattle’s behaviours. The feature extraction processes need to be practical with respect to the demand on computational cost and efficiency. After the features are extracted, machine learning methods can be applied to identify the cattle behaviours. In Table 3, some main contact and non-contact sensor-based cattle behaviour recognition studies are presented.

#### 2.7.1. Contact Sensor-Based Approaches

Contact sensor-based approaches mainly collect individual animal data through sensors fixed on cattle and recognise behaviours according to animal posture (standing or lying), behavioural activity (walking, resting, grazing, and ruminating), and geolocation [101]. For example, Yin et al. [118] used a wireless sensor monitoring system to capture cattle body temperature, respiratory rate, and movement acceleration parameters; then, they used a K-means clustering algorithm to distinguish cattle behaviours. Barriuso et al. [119] presented a multi-agent architecture based on virtual organisations to help farmers monitor the cattle remotely.

González et al. [80] analyzed grazing cattle data from collar-mounted motion and used GPS sensors to perform automatic and real-time behaviour monitoring with high spatial and temporal resolution. Werner et al. [120] validated the RumiWatchSystem as a research tool for measuring detailed grazing behaviour of cows. To improve cattle welfare monitoring and to reduce the labour requirements, Mattachini et al. [70] proposed an automated lying behaviour measurement method for monitoring lactating dairy cows. In the work of [121], pedometers were used to record the step numbers, and the relationship between cattle step numbers, behavioural estrous parameters, and ovulation time were studied. In that work, it was argued that the pedometer is a promising tool to detect estrus and to predict ovulation. Palmer et al. [122] combined visual observations, tail paint, and radiotelemetry (HeatWatch) for 23 cows’ estrus detection. The results in Gibbons et al. [123] highlighted the complexity of aggressive style of cows during feeding and illustrated that some measures of aggressive feeding behaviour were repeatable within cows. Šimić et al. [124] reported that an enriched environment reduced the occurrence of aggressive behaviour in beef cattle.

More recently, Rahman et al. [104] classified cattle behaviour based on a time series of accelerometer data from collar, halter, and ear tag sensors. Riaboff et al. [107] used 3D accelerometer data to predict the behaviours of dairy cows. Peng et al. [93] developed a recurrent neural network (RNN) with a long short-time memory (LSTM) model to detect and recognise calving-related behaviours using inertial measurement unit (IMU). Shen et al. [109] used a triaxial acceleration sensor as the device for collecting mandibular movement data of dairy cow and divided dairy cow behaviours into three categories: eating, ruminating, and other behaviours. In that work, by using K-nearest neighbour algorithm, the recognition accuracy of eating and ruminating reached 92.8% and 93.7%, respectively.

Although contact sensors might have a high precision, they can cause cattle stress. Moreover, the service life of these detection devices can be affected by factors such as scraping and moisture infiltration. In addition, it is impractical to use contact sensors for scoring group behaviours due to their cost and vulnerability.

#### 2.7.2. Non-Contact Sensor-Based Approach

In recent years, a number of non-contact sensor-based approaches have been proposed for undertaking behaviour monitoring and recognition. As non-contact sensors can continuously operate without operator involvement, it is generally believed that they have the potential to assess animal behaviour more quantitatively under a predetermined process that does not change greatly [125,126]. For these reasons, vision/LiDAR-based animal behaviour recognition methods have attracted a lot of attention in the literature [69].

For example, Huang et al. [127] investigated cattle body dimension reconstruction with transfer learning from LiDAR measurements. Gao et al. [128] extracted cattle for moving behaviour tracking and recognition using a dynamic analysis. Gu et al. [112] used minimum bounding box and contour mapping to identify cattle behaviour, and hoof or back characteristics. Meunier et al. [129] integrated a number of image analysis techniques to help determine cows’ main activities (except drinking behaviour). In some other studies, 2D and 3D cameras have been utilised to quantify how much feed was consumed by an individual animal [88,130]. In general, 2D camera monitoring realises behaviour clarification based on shape and colour features, while 3D cameras are more accurate in distinguishing between behaviour using 3D motion detection during feeding and drinking times.

Porto et al. [111] modeled and verified feeding and standing behaviour detection in dairy cows by designing a method based on the Viola–Jones algorithm and a multi-camera video recording system. The sensitivity of this system to feeding and standing behaviours was about 0.87 and 0.86, respectively. Guo et al. [114] used region geometry (for example, inter-frame difference and background subtraction), optical flow characteristics and support vector machine to recognise cow mounting behaviour, achieving a recognition accuracy of 0.98 with 30 videos. Tracking the animal around its enclosure can also lead to the discovery of other important information such as the time taken at the feeder or drinker and can help optimise farm decisions, e.g., the number of feed stations or space requirements [125].

Additionally, sound recognition-based cattle behaviour recognition approaches have also attracted some attention in the cattle industry. Nunes et al. [131] trained a recurrent neural network (RNN) with a long short-term memory (LSTM) layer to detect and distinguish cattle behaviours via chews, bites, and noise. Jung et al. [132] proposed deep learning-based cattle vocal classification model and real-time livestock monitoring system with noise filtering. The proposed approach achieved 81.96% accuracy after the sound filtering. Meen et al. [133] reported a potential welfare monitoring system that observes the vocalisations and behaviours of Holstein Friesian cattle using audio and video recordings. Röttgen et al. [134] reported that the vocalisation rate is a suitable indicator used to confirm a cattle’s estrus status, and it was suggested that the status of the cattle can be monitored through voice analysis. Chelotti et al. [135] estimated grazing and rumination bouts using acoustic signals in grazing cattle and achieved 0.75 F1-scores for both activities. However, how to effectively acquire sound and to accurately determine this information in a livestock facility is still a challenge.

Apart from the above, the potential to identify welfare-compromised animals through motion characteristics or spatial characteristics can has also been explored. Fuentes et al. [77] extracted temporal-context features (3D-CNN) and motion information (optical flow) from videos, achieving 78.8% recognition for 15 different hierarchical behaviours. Yin et al. [115] proposed the EfficientNet-LSTM model to extract spatial feature for the recognition of cows’ motion behaviours, which achieved 97.87% behaviour recognition accuracy in the antagonism of environmental robustness. Wu et al. [13] proposed CNN-LSTM (a fusion of convolutional neural network and long short-term memory) for recognising the basic behaviours of a single cow. In the former work, the experimental results illustrated that the precision of the proposed algorithm for the recognition of five behaviours ranged from 0.958 to 0.995, that the recall ranged from 0.950 to 0.985, and that the specificity ranged from 0.974 to 0.991.

### 2.8. Cattle Behavioural Change Detection and Quantification

Although comprehensive knowledge of the characteristics of the behavioural activities of animals is fundamental, changes in behavioural activity are also important as they reflect exceptional and probably challenging situations caused by internal or external stimuli [88]. Methods of assessing behavioural changes have emerged in recent years with the development of smart sensors and data analysis techniques.

Actually, behavioural change is a good indicator that can reflect diseases or welfare situations [136]. González et al. [81] used data on the feed intake, feeding time, and number of daily feeder visits to describe and quantify changes in short-term feeding behaviour. Their research showed that the quantification of short-term feeding behaviour is helpful in the early identification of sick cows. Overton et al. [137] recorded dairy cow behavioural patterns using time-lapse video photography and examined factors affecting lying behaviour changes during summer conditions. Butt [138] investigated the influences of seasonality in drylands for space–time dynamics of cattle behaviours based on data from GPS collars.

In addition, MacKay et al. [139] illustrated the links between short-term temperament tests and the longer-term behaviour data in beef steers. Some subtle behavioural changes such as walking speed, frequency of standing episodes, or the amount of food intake may also be regarded as indicators of animal health compromises [88]. However, quantifying variable and complex animal behaviour is challenging, and some subtle changes are hard to detect at early stages. Therefore, leveraging long-term videos to measure and quantify the change in behaviours through automated tracking and analysing is of significant value in health and welfare monitoring.

### 2.9. Limitations of Existing Approaches

Most studies focus on the basic behaviours of a single animal such as walking, standing, lying, feeding, and drinking. There are few research on other advanced and group behaviours such as rumination, limping, reproduction, and aggression [79]. In large farms, group or interaction behaviours are also important for animal welfare and the corresponding management. Meanwhile, some tiny behaviours such as limping is part of basic walking behaviours, which is difficult to detect using a general network [13]. In terms of behavioural analysis, environmental conditions are prone to be ignored. Actually, environmental conditions such as temperature, humanity, and carbon dioxide density affect the cattle’s activity and motion behaviours [124].

On the other hand, the majority of the abovementioned behaviour recognition methods require high-definition videos, which may limit their practicability in complex environments such as the low image quality of farm cameras, night, and rainy days. Additionally, quantifying variable and complex animal behaviours based on video data is challenging. The instances of a given behaviour must be recorded and analysed to detect changes with statistical analysis. However, it is time-consuming and often accompanied by error. Additionally, some small behavioural changes are difficult to detect using visual data. All of these severely limit the quantification of animal behaviour changes [77]. It is believed that computer vision combining motion sensor systems could achieve a more economical and accurate behavioural analysis system.

Moreover, the majority of existing monitoring techniques used ground-level sensors such as smart ear tags, camera traps [140], and infrared thermal cameras. Hence, these approaches have limitations in relatively large geographic areas with complex terrains. Remotely sensed imagery can be used to identify dead or live animals with poor mobility by tracking the movement of the animals. This could be a potential alternative and could complement ground-based animal monitoring [141]. The use of quadcopter or satellite data in conjunction with machine learning algorithms is likely to become an emerging and promising direction that can revolutionise livestock management.

## 3. Challenges and Future Research Trends

Based on the above literature review and the livestock development requirements, some main challenges of lameness detection and behaviour recognition are summarised in the following:

(1) Lack of high-quality public data and data fusion methods: Machine learning methods rely on large-scale data to train a favorable model. However, considering the high values of data and the issues of ownership, security, and confidentiality, farms and other commercial entities seldom release their collected data into the public domain [142]. In addition, complex datasets generated from different sources, such as images and motion information, may fail to compensate for functions due to unknown interactions across multiple variables.

(2) Demand for smart management systems: Various sensor data and information could be used to support farm-level decision making, but few management systems could be used to deal with the complex and large-scale data in a broader geographic contexts. How to establish a production and cost management system and how to use this to balance economic and non-economic values from emerging technologies remain challenge to be explored [143].

(3) Lack of commercial availability: The data in laboratory research and production practice have been in a state of disconnect, and actual production is still lacking. Given the techniques used, the performance of the new proposed systems reviewed would be questionable if applied outside the laboratory environment. There is a lack of effective tools to widely use existing data, knowledge, and models [10]. Therefore, a practical system would need to be designed to satisfy applications in a commercial farm environment.

With the development and maturity of various smart sensors, big data, and artificial intelligence, precision livestock farming will develop in the direction of standardisation, large scales, and intelligence with the support of modern equipment. Based on the above review of the research in lameness detection and behaviour recognition, the future research opportunities are discussed in the following:

(1) Animal pose estimation and behaviour changes detection: Pose estimation could help to ensure that cattle in abnormal conditions can be identified on time, hence reducing the possibility of infection and improving the quality of dairy or meat products. In addition, perceived animal’s behaviour changes can provide a basis for automatic determination of its health status, accurate breeding, and other fields. However, research on pose estimation and behaviour change detection is still in its infancy. Advanced pose estimation, behavioural models, and detection theories and methods are important and are desired for future PLF development.

(2) Livestock growth model and intelligent decision support system: Based on big data, perception technology, automatic control technology, and livestock breeding technology, the whole life cycle of an animal can be monitored and analysed. By analysing and processing massive amounts of animal data and information, livestock growth models can be constructed to achieve fine control of livestock and to maximise the benefits of farmers. Meanwhile, decision support systems exploring the trade-off among conflict objectives and offering farmers feasible solutions are desirable. Such systems should consider various data sources, including economic (farm income, profit, and gross domestic product), social (public support subsidy and farm employment), animal welfare and health (body condition, weight, behaviour, reproduction, and growth), and environmental indicators (soil cover, nitrogen, pesticide, and energy). Based on these indicators, an intelligent growth model can be used to assess the trade-off among economic–social–environmental objectives.

(3) New strategies for environmental regulation based on livestock welfare and production performance: Environmental conditions have a significant influence on animal growth rate, behaviours, health status, and productivity. Creating a comfortable growth and production environment for livestock is not only related to the welfare and health of the livestock itself but also closely related to the quality of livestock products, food safety, and economic benefits of the farm. It is necessary to monitor the dynamic changes in the ecological environment parameters in real time. Based on animal behaviour changes, nutrition, growth, and health status, regulatory decisions for the fine control of environmental dynamics and fine feeding of livestock can be made.

(4) The development of more advanced livestock monitoring equipment: It is desirable to develop intelligent equipment and production process robots with embedded perception and intelligent control, from breeding stocks to commercial stock. Industrial applications of intelligent breeding equipment, especially robots, should be combined with breeding modes and livestock facilities in order to improve the overall process efficiency. At the same time, it will also be challenging and rewarding to study animal physiology, growth, and behaviour for better mutual adaptation of equipment and animals and to improve the welfare of animals.

## 4. Conclusions

The global livestock industry has been developing in the direction of standardisation, large scales, and intelligence. Intelligent perception for cattle monitoring is the key to the development of precision livestock farming. The low cost, high efficiency, safety, and sustainability of a large-scale livestock industry can be promoted through the acquisition, processing, analysis, and application of information on cattle welfare and behaviour. Cattle lameness and behaviour are two important indicators for the determination of diseases and health status, early and real-time detection of normal behaviours (e.g., feeding and drinking), and abnormal behaviours (e.g., aggression).

Hence, monitoring cattle lameness and behaviour can reduce the cost of animal production, reduce losses from diseases and mortality, and improve the efficiency of livestock management. In this paper, we conducted a comprehensive survey of intelligent perception for cattle lameness detection and behaviour analysis in the precision livestock farming domain. It is our anticipation that contactless, automated, real-time, and continuous detection will play an important role in PLF. Based on the literature review, we have also discussed the emerging future research trends. Our aim and hope is that this survey will assisst researchers in the field of precision livestock farming, especially in solving various livestock problems involving monitoring cattle welfare and productivity.

## Figures and Tables

**Table 2:** Main cattle behaviour descriptions.

Behaviour	Description
Grazing	Head is placed in or over feed or pasture, while cattle searches, masticates, or sorts the feed (silage) or pasture
Exploring	Head is in close proximity to or in contact with the ground, using the nose to detect smells or food
Grooming	Turns head towards abdomen with a stretched neck, using their tongue to groom the body
Mounting	Animal climbs on any part of the body or head of another animal
Ruminating	The cow regurgitates feed, or swallows masticated feed and regurgitates it
Lying	The cow lies in any position except flat on its side
Walking	The position of the body and four legs changes, with the head and neck not moving
Standing	The cow stands on all four legs with its head erect and without swinging its head from side to side
Aggressive	Causes actual or potential harm (e.g., threat) to other animals

**Table 3:** Main studies on cattle behaviour recognition.

Work	Sensor	Behaviour Type	Feature	Model	Automation Level	Average Accuracy
Contact sensor based approach						
Martiskainen et al. [97]	3D accelerometer	standing, lying, ruminating, feeding, normal, lame walking, lying down, and standing up	Statistical features	SVM	low	94.50%
Tani et al. [98]	single-axis accelerator	chewing	sound spectrogram	pattern matching	low	over 90.0%
González et al. [80]	GPS and 3D accelerometers	foraging, ruminating, traveling, resting, and others	Statistical features	Statistical analysis	medium	90.5%
Smith et al. [99]	motion collars	grazing, walking, ruminating, resting, and others	head position and motion intensity	Binary time series classifiers	medium	82.25%
Williams et al. [100]	GPS	grazing, resting, and walking	statistical features	machine learning	medium	85.0%
Williams et al. [101]	GPS data	grazing, resting, and walking	behaviour-labelled GPS data	hidden Markov model	medium	94.0%
Andriamandroso et al. [102]	IMU	grass intake and ruminating	statistical features	two-step discrimination tree	low	92.0%
Wang et al. [103]	3D accelerometer	standing, lying, normal walking, active walking, standing up, and lying down	statistical features	binary decision-tree	medium	76.47%
Rahman et al. [104]	3D accelerometer	grazing, standing, or ruminating	statistical features	Stratified Cross Validation	medium	91.2%
Achour et al. [105]	IMU	lying, standing, lying down, standing up, walking, and stationary behaviours	statistical features	Finite Mixture Models	medium	99.0%
Peng et al. [106]	IMU	feeding, lying, ruminating licking salt, moving, social licking, and head butting	motion data	LSTM-RNN model	medium	88.65%
Riaboff et al. [107]	3D accelerometer	grazing, walking lying, and standing	statistical features	decision tree	medium	95.0%
Williams et al. [108]	3D accelerometer	drinking	statistical features	accelerometer algorithm	medium	95.0%
Peng et al. [93]	IMU	ruminating (lying), ruminating (standing), lying normal, standing normal, feeding, lying final, and standing final	deep learning features	LSTM-RNN	high	77.56%
Shen et al. [109]	3D accelerometer	eating, ruminating, and other behaviours	time/frequency-domain features	K-nearest neighbour	high	93.25%
Tran et al. [110]	3D accelerometer	walking, feeding, lying, and standing	statistical features	Random Forest algorithm	high	94.75%
Non-contact sensor-based approach						
Tsai and Huang [96]	camera	estrus and mating behaviour	changes of moving object lengths	motion analysis	medium	99.67%
Dutta et al. [82]	camera	grazing, ruminating, resting, walking, and other	sensor data and behaviour observations	bagging ensemble classification	medium	96%
Porto et al. [111]	camera	feeding and standing	image detectors	Viola–Jones algorithm	medium	86.5%
Gu et al. [112]	camera	estrus and hoof disease behaviours	minimum bounding box area	Dynamic Analysis	medium	83.40%
Ahn et al. [113]	camera	mounting, walking, running, tail wagging, and foot stamping	motion history image feature	SVM	medium	82.83%
Guo et al. [114]	camera	mounting behaviour	geometric and optical flow characteristics	SVM	medium	90.9%
Yin et al. [115]	camera	lying, standing, walking, drinking, and feeding	visual features	EfficientNet-LSTM	high	97.87%
Achour et al. [116]	camera	standing and feeding	visual features	CNN	high	92.00%
Fuentes et al. [77]	camera	15 types: standing, lying, lying, and others	3D-CNN features	deep learning	high	78.80%
Wu et al. [13]	camera	drinking, ruminating, walking, standing, and lying	visual features	CNN-LSTM	high	97.60%
Guo et al. [117]	camera	exploring, feeding, grooming, standing, and walking	visual features	BiGUR-attention	high	over 82%

Note: SVM means support vector machine; LSTM means Long Short Term Memory network; RNN means Recurrent Neural Network; BiGRU is short for Bidirectional Gated Recurrent Unit.

## Data Availability

No new data were created or analyzed in this study. Data sharing is not applicable to this article.
